# Combining data‐derived priors with postrelease monitoring data to predict persistence of reintroduced populations

**DOI:** 10.1002/ece3.4060

**Published:** 2018-05-22

**Authors:** Faline M. Drummond, Tim G. Lovegrove, Doug P. Armstrong

**Affiliations:** ^1^ Wildlife Ecology Group Massey University Palmerston North New Zealand; ^2^ Biodiversity Unit Auckland Council Auckland New Zealand

**Keywords:** Bayesian inference, fecundity, population modeling, population viability, prior information, reintroduction, survival analysis, translocation

## Abstract

Monitoring is an essential part of reintroduction programs, but many years of data may be needed to obtain reliable population projections. This duration can potentially be reduced by incorporating prior information on expected vital rates (survival and fecundity) when making inferences from monitoring data. The prior distributions for these parameters can be derived from data for previous reintroductions, but it is important to account for site‐to‐site variation. We evaluated whether such informative priors improved our ability to estimate the finite rate of increase (λ) of the North Island robin (*Petroica longipes*) population reintroduced to Tawharanui Regional Park, New Zealand. We assessed how precision improved with each year of postrelease data added, comparing models that used informative or uninformative priors. The population grew from about 22 to 80 individuals from 2007 to 2016, with λ estimated to be 1.23 if density dependence was included in the model and 1.13 otherwise. Under either model, 7 years of data were required before the lower 95% credible limit for λ was > 1, giving confidence that the population would persist. The informative priors did not reduce this requirement. Data‐derived priors are useful before reintroduction because they allow λ to be estimated in advance. However, in the case examined here, the value of the priors was overwhelmed once site‐specific monitoring data became available. The Bayesian method presented is logical for reintroduced populations. It allows prior information (used to inform prerelease decisions) to be integrated with postrelease monitoring. This makes full use of the data for ongoing management decisions. However, if the priors properly account for site‐to‐site variation, they may have little predictive value compared with the site‐specific data. This value will depend on the degree of site‐to‐site variation as well as the quality of the data.

## 1 INTRODUCTION

Species reintroduction is a costly exercise that has historically had a low rate of success (Fischer & Lindenmayer, 2000; Griffith, Scott, Carpenter, & Reed, 1989; Griffiths & Pavajeau, 2008). It is well recognized that reintroductions have suffered from insufficient monitoring, with failure to learn from monitoring probably contributing to the poor success (Griffith et al., 1989; IUCN 2013; Lyles & May, 1987). Increased monitoring has improved the ongoing management of reintroduced populations and helped to guide strategies for future reintroductions (Seddon et al. 2007). It has also facilitated the development of quantitative models to make predictions about population dynamics, which can be used to guide a range of decisions (Converse & Armstrong, 2016).

Models used in reintroduction programs fall into two main types: those used to predict site suitability prerelease and those used to predict population persistence postrelease (Chauvenet, Parlato, Gedir, & Armstrong, 2015; Converse, Moore, & Armstrong, 2013). The latter models typically use data collected on vital rates (survival and fecundity), with data collection starting at the time of release. They are therefore similar to other models used for population viability analysis (Beissinger & Westphal, 1998), but with greater emphasis on transient dynamics associated with postrelease effects, initially small population sizes, and unstable sex and age structures (Burgman, Ferson, & Lindenmayer, 1994; McCallum, 1994). Such models are famously “data hungry,” meaning large data sets are needed to make precise predictions, and therefore, most predictions are highly uncertain (Possingham, Lindenmayer, & Norton, 1993). Quantifying this uncertainty should therefore be considered essential when making management decisions for populations (Beissinger & Westphal, 1998).

The treatment of uncertainty in population projections has advanced markedly in the last 20 years, mainly due to the advent of Bayesian hierarchical modeling (Clark, 2005; King, Morgan, Gimenez, & Brooks, 2009; Link & Barker, 2010). Bayesian hierarchical modeling is a flexible approach that potentially allows complex variation to be modeled even with relatively small data sets, allowing multiple sources of uncertainty to be quantified (Clark & Gelfand, 2006). This method therefore lends itself to reintroductions as they often involve small data sets and multiple uncertainties (Converse & Armstrong, 2016). However, reintroduction programs have not exploited the most basic feature of Bayesian modeling because prerelease and postrelease inferences are typically disconnected.

The basic concept of Bayesian inference is that prior knowledge and new data can be combined using a model to produce posterior knowledge (Link & Barker, 2010). The prior knowledge can potentially take the form of expert judgment (Martin et al., 2012). However, it may also be possible to obtain data‐derived priors through quantitative analysis of previous data (Morris, Vesk, McCarthy, Bunyavejchewin, & Baker, 2015). Although applied ecologists are always influenced by data from previous studies, they usually only incorporate this information implicitly in their sampling designs or discussions (McCarthy & Masters, 2005). The advantage of explicitly incorporating prior information is that this may reduce the amount of data needed before useful predictions can be made (McCarthy & Masters, 2005; Morris et al., 2015), potentially reducing the need for expensive long‐term monitoring (Likens, 1983; Taylor, 1989).

Reintroduction programs naturally lend themselves to Bayesian inference because the decision to undertake a reintroduction must be based on some form of prior knowledge (IUCN 2013), which can then be updated based on postrelease data. Such inference lends itself to adaptive management, both for ongoing management of reintroduced populations and for making future decisions about proposed reintroductions (McCarthy, Armstrong, & Runge, 2012). However, as in other fields of applied ecology (Morris et al., 2015), such inference is not currently part of the reintroduction practioner's “toolbox”. Parameter estimates from previous research have been used to make prior predictions about reintroduced populations (e.g., South, Rushton, & Macdonald, 2000), but this has not involved deriving prior distributions that account for uncertainty. Gedir, Thorne, Brider, and Armstrong (2013) explicitly used Bayesian inference when modeling dynamics of a reintroduced population, with prior data for vital rates obtained from two previous reintroductions. However, because they only had data for two previous sites, they were not able to derive prior distributions that accounted for site‐to‐site variation. Canessa et al. (2016) used Bayesian inference to model survival rates of a reintroduced population, with a combination of data and expert judgment used to obtain the priors.

We report the first case study illustrating how fully data‐derived priors can be combined with postrelease monitoring data when making predictions for reintroduced populations. Parlato and Armstrong (2012) earlier showed how data on vital rates for multiple reintroduced populations of North Island (NI) robin (*Petroica longipes*) populations could be integrated using Bayesian hierarchical modeling, allowing random site‐to‐site variation to be accounted for. This meant that prior distributions could be derived for the finite rate of increase (λ) expected at proposed reintroductions, meaning that site selection could be improved over time through passive adaptive management (McCarthy et al., 2012). Here, we extend this approach by showing how such data‐derived priors can be progressively updated as postrelease data are collected at a new reintroduction site, potentially facilitating ongoing management decisions at that site. To test the usefulness of the priors, we compared how the precision of the estimated finite rate of increase (λ) increased over time if the data‐derived priors were or were not used, to determine how many years of data were required to be confident of population persistence.

## 2 MATERIALS AND METHODS

### 2.1 Species and study area

**Figure 1:**
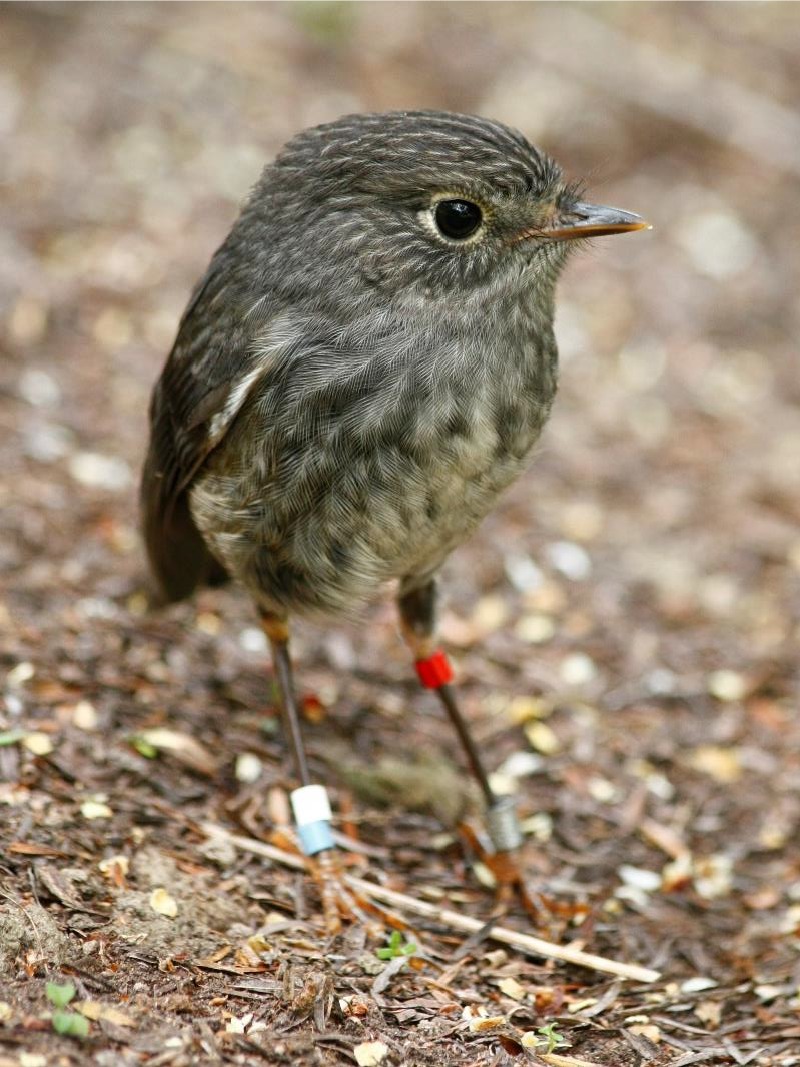
A juvenile North Island robin (*Petroica longipes)* at Tawharanui Regional Park in 2016. Photograph credit: Jonas Kotlarz

The NI robin (Figure 1) is a small (26–32 g) forest passerine endemic to New Zealand (Higgins & Peter, 2002). NI robins are mainly insectivorous, monogamous, territorial, and sedentary, with adults rarely leaving their territories once established. Females can lay up to three clutches from September to February. Juveniles usually undergo a dispersal phase shortly after fledgling, and if they survive the winter, become sexually mature by the start of the following breeding season.

NI robins were widespread throughout the North Island and nearby offshore islands at the time of European settlement, but disappeared from most of their original range following forest clearance and introduction of mammalian predators. They have now been reintroduced to many sites where mammalian predators have been controlled or eradicated, with these reintroductions having mixed success (Miskelly & Powlesland, 2013; Parlato & Armstrong, 2012). This scenario is ideal for assessing the usefulness of data‐derived priors because multiple reintroductions have taken place, postrelease data have been collected using fairly consistent methodology, the biology and threats are well understood, but there remains considerable uncertainty about whether many reintroductions will be successful or not.

The reintroduction featured here was to Tawharanui Regional Park, a 558 ha peninsular reserve approximately 80 km north of Auckland, New Zealand. An aerial poison operation, ongoing intensive predator control, and an open ended 2.7 km predator exclusion fence installed in 2004 have effectively eradicated all exotic mammal species except mice (*Mus musculus*) and rabbits (*Oryctolagus cuniculus*). There is ongoing monitoring for predator incursions (Maitland, 2011). In March 2007, 21 NI robins (14 male, 7 female) were translocated from Tiritiri Matangi Island to Tawharanui. A further four females were translocated from Puhoi near Wenderholm in July‐August 2007. NI robins now occupy approximately 120 ha of Tawharanui.

### 2.2 Data collection

Annual surveys were carried out in September (the start of the breeding season) from 2007 to 2016 to generate data on survival of color‐banded individuals. Fecundity data were obtained through weekly checks of known robin pairs. These checks consisted of recording the breeding status (nonbreeding, number of eggs, chicks, and fledglings) of each nest and generating data on the number of young fledged over the season by each pair.

Pairs were usually located by walking through the territory, but playback calls were occasionally used when pairs were not easily found. Nests were located by feeding mealworms (*Tenebrio molitor*) to the birds. During incubation, the male would call the female off the nest, or if the chicks had hatched, either parent would take mealworms to the nest. Nestlings were typically banded 9–12 days after hatching, with 660 chicks being banded in the nest between 2007 and 2016. There were only a few instances (4), where young birds were caught using a claptrap or hand net and banded as fledglings. It was not possible to color band all chicks every year as some nests were inaccessible and the occasional nest was missed during monitoring. However, the number of unbanded birds has remained relatively low, and most (> 95%) of the population has always been banded.

Due to the number of pairs increasing (9 in 2007 to 37 in 2016), the nests could not all be monitored through to fledging. To maintain consistency between years, chicks were considered to have fledged if they survived to banding age. The number considered fledged was therefore slightly higher than the number recorded as leaving the nest, but more intensive data collection from the 2015 to 2016 breeding season showed that the proportion of chicks that died between banding and fledging was quite small (< 1%).

### 2.3 Modeling

We modeled the data using OpenBUGS version 3.2.3 which uses Markov Chain Monte Carlo (MCMC) techniques to fit Bayesian hierarchical models (Spiegelhalter, Thomas, Best, & Lunn, 2014). This approach allows multiple random effects and also facilitated an integrated modeling approach where all data are modeled simultaneously to generate population projections that fully account for parameter uncertainty and covariance (Schaub & Abadi, 2011). Models were run for up to 50,000 iterations with an initial burn in of 5,000 samples after checking convergence by examining the chains and autocorrelation plots.

We generated informative priors using Parlato and Armstrong's (2012) model, which integrated demographic data from 10 reintroduction sites to predict what would happen at a proposed reintroduction site. We adapted this model by removing the two years of Tawharanui data that had been originally included and used the model to generate prior distributions for four parameters: 1) mean fecundity (number of fledglings per female per year), 2) random effect of individual female on fecundity, 3) probability of an adult surviving one year, and 4) the probability of a juvenile surviving from fledgling to adulthood (Appendix S1). The priors for the survival parameters are specific to peninsular sites, as Parlato and Armstrong (2012) found apparent survival of juveniles to be lower on peninsular than nonpeninsular sites, and the significance of this effect was retained when the Tawharanui data were removed. They hypothesized that apparent juvenile survival was lower at peninsular sites because juveniles dispersed along forest edges into unprotected habitat outside the site.

We initially modeled the Tawharanui data using uninformative priors (Appendix S1) and started by examining the effects of all variables we believed may affect survival or fecundity. Priors were taken to be normally distributed for main parameters (regression coefficients) and uniformly distributed for hyperparameters (standard deviation of random effects). We then reduced the model by removing fixed effects if their 95% credible intervals included zero, and removing random effects if the lower portions of their posterior distributions were concentrated near zero (Kéry & Schaub, 2012).

Fecundity was modeled with a log link function and Poisson error distribution. The full fecundity model included a fixed effect of density and two random effects, one for the individual female and the other for year. Due to high adult survival (see below), most breeding females occurred over multiple years in the fecundity data set. Including the random female effect allowed variation among individual females and ensured that the results were robust to potential pseudoreplication. Age was not considered, as previous studies suggest that age of female robins does not affect their fecundity (Dimond & Armstrong, 2007).

Survival was modeled using a state‐space formulation of the Cormack–Jolly–Seber (CJS) model (Kéry & Schaub, 2012). Both survival and resighting were modeled with logit link functions and Bernoulli error distributions. Survival surveys were conducted annually at the start of each breeding season, and the difference in the time interval between the translocation and first annual survival survey (6 months) was corrected for. The full survival model included fixed effects of age (adult vs. juvenile), sex (adults only), and translocation (first 6 months’ vs. subsequent adult survival), as well as a fixed effect for density on juvenile survival. An effect of banding age was also included to correct for the higher survival probability expected in the four juveniles banded as fledglings, compared with those banded in the nest. Random annual variation on juvenile survival was included to allow for changes in survival over time due to weather and other factors.

Annual abundance was also estimated as this enabled us to observe how population size had changed over time and model density dependence in survival and fecundity. There were two components to estimating yearly abundance: estimating the number of banded birds alive at each survival survey based on the CJS model and estimating the number of unbanded birds present. We assumed that detection probability was equal for banded and unbanded birds. We obtained separate estimates for males and females and then combined these to estimate the total.

The reduced model was used to derive the finite rate of increase, which is given by:

$$\lambda =s_{a}+\frac{1}{2}s_{j}f,$$

where *s*_a_ is annual adult survival probability, *f* is the mean number of fledglings per female per year and *s*_j_ is the apparent juvenile survival probability (probability of both surviving from fledging until adulthood and staying at Tawharanui). We generated λ with both informative priors and uninformative priors. For both approaches, we added the Tawharanui data one year at a time to assess how the precision of the λ estimate and usefulness of the priors changed with the amount of data available.

The code for the reduced model is presented in Appendix S2, and the data are presented in Appendix S3.

## 3 RESULTS

### 3.1 Abundance

**Figure 2:**
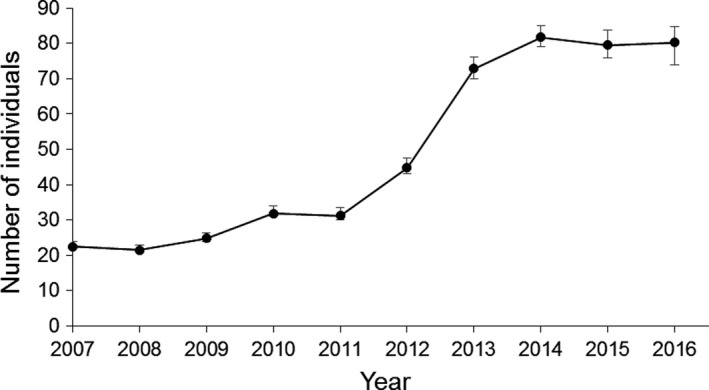
Growth of the North Island robin population reintroduced to Tawharanui. Points show estimated numbers at the start of each breeding season, with 95% credible intervals

The Tawharanui population increased from 22 birds in 2007 to about 80 birds in 2016 (Figure 2). There was a slow increase from 2007 to 2011 followed by a more rapid increase after the 2011 breeding season, and the population size remained relatively constant from 2014 to 2016.

### 3.2 Fecundity

**Figure 3:**
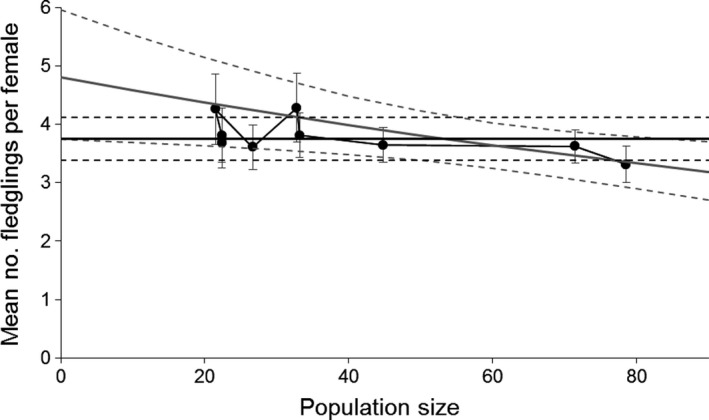
Changes in mean fecundity (fledglings per female) of North Island robins at Tawharanui Regional Park in relation to breeding population size. The dots and error bars show annual estimates and standard errors. The gray curve shows the estimated relationship between fecundity and density, whereas the black line shows the estimated fecundity if density dependence is excluded from the model. Dotted lines show 95% credible intervals. The models shown here had uninformative priors for all parameters, but the results are very similar with informative priors

There was a trend for fecundity to decrease as population size increased, but this effect was ambiguous (Figure 3, Appendix S4). Thus, there were two fecundity models with similar support: a “constant” model and a “density” model. Under the constant model, an average female was estimated to have 3.8 fledglings, whereas under the density model, this was expected to decline from 4.8 to 3.3 fledglings as the population grew from 0 to 80. While there was random variation in fecundity among individual females, there was no evidence of random variation among years (Appendix S4).

**Figure 4:**
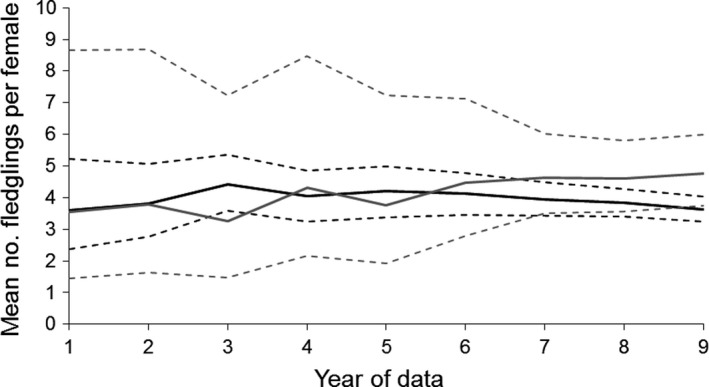
Changes in the estimated mean number of fledglings per female for North Island robins at Tawharanui Regional Park, as a function of the number of years of postrelease monitoring data. Black lines show estimates of the intercept based on a constant model, and gray lines show estimates of the intercept based on the density‐dependent model. Dotted lines show 95% credible intervals. Both models had uninformative priors for all parameters

The precision of fecundity estimates depended on both the model and amount of data available (Figure 4). The “constant” model was supported for the first 7 years, as it took 8 years before the posterior distribution for the density effect to be narrow enough that it no longer included 0. The ambiguity of the density effect for the final two years means that it was unclear which model gave the best fecundity estimate (Figure 4).

### 3.3 Survival

**Figure 5:**
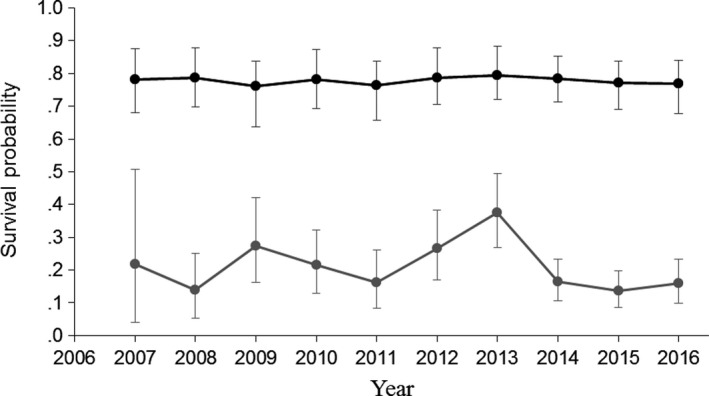
Estimated annual survival of adult (black) and juvenile (gray) North Island robins at Tawharanui under a model with random year effects, with uninformative priors used for all parameters. Error bars show 95% credible intervals

Adult survival was relatively constant (ca. 0.78) over time (Figure 5, Appendix S4), so we removed the random time effect on adult survival from the model. The fixed sex and translocation effects were also removed, as there was no evidence for a difference in survival between males and females, or between survival over the first 6 months and later adult survival (Appendix S4). Juvenile survival was considerably lower than adult survival and varied from 0.14 to 0.38 among years (Figure 5). There was no evidence that density dependence caused this variation, as the 95% credible interval for the density effect of juvenile survival was centered near 0 (Appendix S4), and there was no decrease in juvenile survival over time (Figure 5). The probability of resighting a bird at each survey was 0.90, and this was constrained to be constant over time as the 95% confidence interval for the random year effect was centered near 0 (Appendix S4).

**Figure 6:**
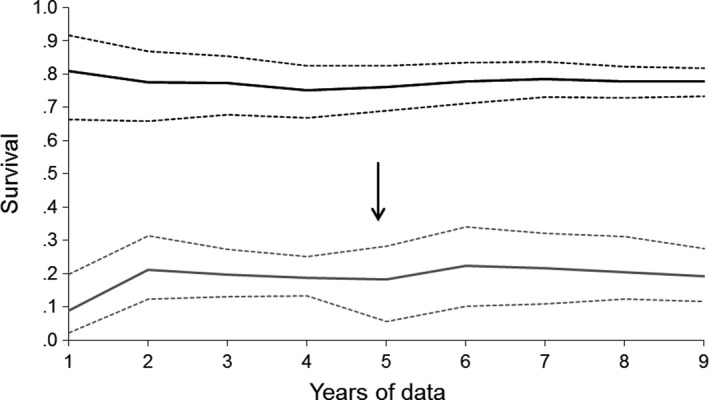
Changes in the estimated survival probabilities of adult (black) and juvenile (gray) North Island robins as a function of the number of years of postrelease monitoring data. Solid lines show estimates, and dotted lines show 95% credible intervals. All variables in the reduced model fitted to all 9 years of data are included, except for the random year effect on juvenile survival which was added when 5 years of data were available (black arrow). Uninformative priors were used for all parameters

The precision of adult survival estimates increased as more data became available (Figure 6). For juvenile survival, random annual variation became apparent after 5 years of data were available (posterior distribution no longer concentrated near zero), so the model selected was changed at this stage, increasing the accuracy of the estimates but reducing precision (Figure 6).

### 3.4 Population growth

Because it was unclear which was the best fecundity model, we estimated λ under models that did or did not include density dependence in fecundity. After 9 years of monitoring, the model with no density dependence gave a λ estimate of 1.13, with a lower 95% credible limit near 1 (Table 1). The model including density dependence gave a slightly higher λ estimate of 1.23 at zero density, but the lower credible limit was also near 1 due to the greater standard deviation under this model.

**Table 1:** Finite rate of increase (λ) estimates for the North Island robin population at Tawharanui Regional Park. The first row gives estimates based only on prior data from nine other reintroduction sites. All other rows show estimates after 9 years of monitoring the Tawharanui population, with and without the ambiguous density dependence (DD) in fecundity. Separate estimates are shown for when informative (I) and uninformative (U) priors were used. λ decreases with increasing population density under the density‐dependent model, and the values shown are for 0 density

Tawharanui data	Priors	λ			
		DD	Mean	2.5% CL	97.5% CL
No	I	N/A	1.08	0.76	1.66
Yes	U	No	1.13	0.99	1.30
Yes	I	No	1.11	0.99	1.25
Yes	U	Yes	1.23	1.03	1.48
Yes	I	Yes	1.20	1.02	1.40

**Figure 7:**
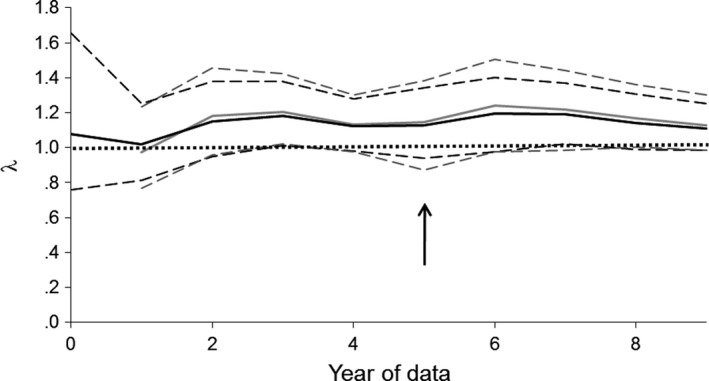
Changes in the estimated finite rate of increase (λ) of the reintroduced North Island robin population at Tawharanui Regional Park as a function of the number of years’ postrelease monitoring data. Black lines show estimates (solid lines) and 95% credible intervals (dotted lines) using informative priors, whereas gray lines show estimates and 95% credible intervals using uninformative priors. The finely dotted line shows a λ value of 1, meaning the population is expected to persist. The arrow shows where random annual variation in juvenile survival was added to the models (Figure 6), resulting in wider credible intervals. The models showed here exclude density dependence in fecundity

The estimated λ was always above 1 but its 95% credible interval was not completely > 1 until 7 years of data had been collected (Figure 7), meaning it was unclear until that stage whether the population was expected to persist. Although the credible intervals appeared to be >1 after 3–4 years, these intervals should be regarded as overprecise because they do not account for the substantial annual variation in juvenile survival revealed by subsequent data.

Informative priors enabled λ to be predicted when no Tawharanui data were available. They subsequently allowed slightly narrower credible intervals, especially when the random year effect on juvenile survival was added to the model at 5 years (λ 7). However, they did not reduce the number of years of data that needed to be collected for the lower 95% credible limit for λ to be > 1.

## 4 DISCUSSION

Models for reintroduced populations are useful for predicting population persistence under current conditions to assess whether future management is likely to be required and for improving predictions for future reintroductions. As for any conservation scenario, the appropriate monitoring and modeling approaches depend on the management problem (Nichols & Williams, 2006). The approach used here was to collect detailed data on vital rates from the time of release and model these in combination with data‐derived priors for previous reintroductions.

Monitoring vital rates are labor‐intensive, and therefore expensive, but are otherwise advantageous in the short term because it provides maximum power to make inferences. Monitoring population trends alone not only has lower power, but can give misleading inferences due to an inability to model transient dynamics associated with sex ratios, age structure and stochasticity, and make unbiased inferences about density dependence (Caswell, 2001). The reintroduced NI robin population at Tawharanui is a good example, as the pattern of increase over time (Figure 2) could be interpreted to mean that the population is tightly regulated with a carrying capacity of about 80. In contrast, analysis of the vital rate data indicated that density dependence was weak or nonexistent at this stage, with a carrying capacity of approximately 200 if density dependence in fecundity is assumed (the population size at which λ declines to 1 based on the parameter estimates in Appendix S4). The observed pattern of increase is therefore entirely attributable to stochasticity.

The focus of our analysis was on determining whether λ was >1 at low density, indicating whether management intervention would be needed for the population to persist. Once growth is ensured, it is sensible to focus on the longer‐term viability of the population which will be affected by its dynamics and regulation (Sarrazin, 2007). One factor to consider is inbreeding, which can potentially reduce vital rates over time if populations remain small (Frankham, Briscoe, & Ballou, 2002). Inbreeding was found to moderately reduce juvenile survival, but has no detectable effect on fecundity, in NI robins on Tiritiri Matangi Island (Jamieson, Tracy, Fletcher, & Armstrong, 2007) which was the source population for the Tawharanui reintroduction. However, effects on population dynamics were expected to be negligible for time frames <100 years (Jamieson, 2011).

The key results of our case study were that λ was indeed found to be > 1 at low density, but that 7 years of monitoring data were needed before we could be confident that this was the case, and this requirement was not reduced by the use of informative priors. These results show that it can be quite difficult to confirm the future persistence of reintroduced populations, even when intensive monitoring has been conducted for several years. They also show the importance of properly translating uncertainty about vital rates into λ estimates (Schaub & Abadi, 2011; Wade, 2002), as there may be considerable risk that a reintroduced population will decline even though the λ estimate is > 1. Finally, they show that informative priors may have negligible effect on this uncertainty.

We caution that these particular results are case‐specific and will depend on several factors. The period of uncertainty will tend to be prolonged if the initial population size is small, λ is close to 1, or there is annual variation in one of more vital rates (i.e., environmental stochasticity), all of which were the cases for the Tawharanui NI robin population. It is not surprising that five years of data were needed before the annual variation in juvenile survival could incorporate, as 5–6 levels are typically needed for hierarchical modeling (Gelman & Hill, 2007). The approach we used here was to exclude annual variation and other random effects until there were sufficient data to estimate them (see Modeling). However, if annual variation is anticipated, a better approach may be to use a weakly informative prior so this variation can be modeled from the outset. Data‐based priors could also be used if available, as was the case for individual variation among females (Appendix S1), but these were not available for annual variation because most of the previous data sets were from short‐term studies (Parlato & Armstrong, 2012).

The degree to which informative priors reduce data requirements will depend on the variances of those priors in relation to the postrelease data collected. In our case study, the relatively high variance in the priors reflected the degree of unexplained site‐to‐site variation in previous NI robin reintroductions (Parlato & Armstrong, 2012). Although postrelease sample sizes were limited by the initially low population size, the data were of high quality due to the intensive monitoring regime. Consequently, the postrelease data quickly “overwhelmed” the priors (Link & Barker, 2010), but this will not always be the case. Gedir et al. (2013) inferred that informative priors saved about one year of postrelease monitoring data, but this may have been an overestimate given that their priors did not incorporate site‐to‐site variation.

Regardless of the amount of postrelease monitoring saved, there are several reasons why we believe that the Bayesian approach should be widely applied to inferences for reintroduced populations. First, quantitative derivation of informative priors provides a more transparent basis for prerelease decisions than the intuitive procedures often followed, and if done well should allow managers to see the uncertainty involved. For example, the 95% credible interval for λ ranged from 0.76 to 1.66 prior to the NI robin reintroduction to Tawharanui, showing that a wide range of outcomes was possible. Second, deriving explicit priors for vital rates allow reintroduction practitioners to test the reliability of their prerelease predictions using postrelease data, allowing adaptive management (McCarthy et al., 2012). Third, explicit priors make it possible to predict the value of postrelease data collected and therefore design monitoring programs strategically (Canessa et al., 2016). Finally, the Bayesian approach is the natural framework for integrating prerelease and postrelease inferences, making full use of the information available (Morris et al., 2015).

The final point depends on the priors being valid, as they may otherwise bias parameter estimates, especially for small data sets. This potential problem is avoided if fully data‐derived priors are used, meaning not only that the prior data are as equally objective as the new data (Morris et al., 2015), but also that the uncertainty involved in extrapolating prior data to a new site is properly accounted for (Parlato & Armstrong, 2012). Although we were able to adopt this approach, this will be difficult with most reintroductions due to lack of high‐quality data for multiple sites, and hence, some degree of expert judgment will be needed to derive priors (Canessa et al., 2016). This is where most of the controversy surrounding Bayesian inference stems from, as the validity of the priors depends on how well the subjectivity of expert judgment is accounted for (Pan & Yontay, 2017; Spiegelhalter, Myles, Jones, & Abrams, 2000). However, recent advances in structured elicitation (Martin et al., 2012; McBride, Fidler, & Burgman, 2012) should allow prior expert judgment of vital rates to play an increasingly important role in reintroduction programs (Converse & Armstrong, 2016).

In conclusion, we advocate increased use of Bayesian frameworks to integrate prerelease and postrelease inferences about reintroduced populations, but caution that prior information should be interpreted carefully. Fully data‐derived priors are ideal, and the research described here illustrates how such priors can be incorporated into models used to predict reintroduction outcomes. However, if priors properly reflect the many uncertainties involved in reintroduction (IUCN 2013), their predictive value may be quickly overwhelmed by postrelease data, which will therefore continue to be invaluable.

## CONFLICT OF INTEREST

None declared.

## AUTHORS’ CONTRIBUTIONS

F.M.D., D.P.A., and T.G.L. conceived the ideas and designed the methodology; T.G.L. and F.M.D. collected the data; F.M.D. and D.P.A. analyzed the data; F.M.D. and D.P.A. led the writing of the manuscript. All authors contributed critically to the drafts and gave final approval for publication.

## DATA ACCESSIBILITY

The data are provided in BUGS format in Appendix S3 (Supporting Information) and in spreadsheet format in the Dryad Digital Repository (details to be added).
