# Supplementary material for: Combining data‐derived priors with postrelease monitoring data to predict persistence of reintroduced populations
Source: Ecol Evol. 2018 May 22;8(12):6183–91. doi: 10.1002/ece3.4060 (PMC6024125; doi:10.1002/ece3.4060)
Supplement: Supplementary file 1 [file ECE3-8-6183-s001.docx]

**Appendix S1.** Informative and uninformative prior distributions for key parameters in the population model for North Island robins reintroduced to Tawharanui Regional Park. The informative priors are based on data for 9 robin reintroductions to other sites. The normal distributions (*N*) show means and standard deviations, and the uniform distributions (*U*) show the ranges.

| **Parameter** | **Informative** | **Uninformative** |
| --- | --- | --- |
| a.f | *N*(1.27, 0.38) | *N*(0, 3.16) |
| sd.fem.f | *N*(0.23, 0.09) | *U*(0, 1) |
| a.phi | *N*(-1.76, 0.60) | *N*(0, 3.16) |
| b.age.phi | *N*(3.05, 0.95) | *N*(0, 3.16) |

*a.f, intercept term for log of mean fecundity (number of fledglings per female); sd.fem.f, standard deviation for random effect of individual female on log fecundity; a.phi, intercept term for logit of probability of a juvenile surviving from fledgling to adulthood and staying at Tawharanui; b.age.phi, difference between logit annual adult female survival and logit juvenile survival.

**Appendix S2.** OpenBUGS code for modelling abundance, fecundity, survival, and finite rate of increase (λ) of the North Island robin population reintroduced to Tawharanui Regional Park. This shows a reduced model following initial variable selection (see Appendix S4 for variables removed from full model).

Model {

# INFORMATIVE (I) AND UNINFORMATIVE (U) PRIORS

# comment out ones that don't apply

# Fecundity parameters (f = mean number of fledglings per female per year)

a.f ~ dnorm(0,0.1) # intercept for log mean fecundity (U)

# a.f ~ dnorm(1.268,6.932) # intercept for log mean fecundity (I)

b.d.f ~ dnorm(0,1) # effect of population size on log (f) (U)

sd.fem.f ~ dunif(0,1) # SD in log(f) among individual females (U)

# sd.fem.f ~ dnorm(0.227,116.6) I(0,) # SD in log(f) among individual females (I)

tau.fem <- pow(sd.fem.f,-2) # next 2 lines convert SDs to precisions

# Survival and re-sighting parameters (sj = juvenile survival prob, sa = adult survival prob)

a.phi ~ dnorm(0, 0.1) # intercept for logit(sj) (U)

# a.phi ~ dnorm(-1.76, 2.75) # intercept for logit(sj) (I)

b.age.phi ~ dnorm(0, 0.1) I(0,) # effect of being adult (U)

# b.age.phi ~ dnorm(3.051, 1.110) # effect of being adult (I)

b.bfl ~ dnorm(0, 0.1) I(0,b.age.phi) # effect of banding at fledgling stage (U)

sd.yr.juvphi ~ dunif(0,2) # sd in logit(sj) among time intervals (U)

tau.yr.juvphi<-pow(sd.yr.juvphi, -2) # convert to precision

p.male[1] ~ dunif(0,1) # probability of recruit being male (U)

p.male[2] ~ dunif(0,1) # probability of translocated bird being male (U)

a.p ~ dnorm(0, 0.1) # mean logit(p), where p = re-sighting prob (U)

logit(p) <- a.p

# ASSIGN INDIVIDUAL RANDOM EFFECTS ON FECUNDITY

for (i in 1:n.fem) {

re.fem[i] ~ dnorm(0,tau.fem)

}

# MODEL NUMBER OF FLEDGLINGS PER FEMALE PER YEAR

for (i in 1:n.f.obs) { # for each fecundity observation

fl[i] ~ dpois(mu[i]) # sample observed no. fledged

log(mu[i]) <- a.f+re.fem[fem[i]]+b.d.f*D[year[i]] # expected no. fledglings at current density

}

# ASSIGN RANDOM YEAR EFFECTS ON JUVENILE SURVIVAL

for (j in 2:n.surveys) {

re.yr.juvphi[j] ~ dnorm(0, tau.yr.juvphi)

}

# MODEL SURVIVAL

for (i in 1:n.ind) { # for each individual...

sex[i] ~ dbern(p.male[trans[i]+1]) # model unknown sexes

for (j in 1:first[i]) { # this is used for counting number adults (see below)

alive.ad[i,j] <- 0

}

alive[i, first[i]] <- 1 # each individual known alive on first encounter

for (j in first[i]+1:n.surveys) { # for each subsequent survey....

age[i,j] <- max(step(j-first[i]-2),trans[i]) # whether bird was an adult over interval

seen[i,j] ~ dbern(psight[i,j]) # whether individual is seen

psight[i,j] <- p*alive[i,j] # probability of individual being seen

alive[i,j] ~ dbern(palive[i,j]) # whether individual is alive

alive.ad[i,j] <- alive[i,j] # whether individual is a live adult

palive[i,j] <- phi.int[i,j]*alive[i,j-1] # probability individual is alive

phi.int[i,j] <- pow(phi[i,j],int[j]) # probability of surviving the last interval

logit(phi[i,j]) <- a.phi+b.age.phi*age[i,j]+b.bfl*bfl[i]*(1-age[i,j])+re.yr.juvphi[j]*(1-age[i,j])

# survival probability

}

}

# MODEL ABUNDANCE

N[2] <- sum(alive.ad[,2]) # no. banded adults from survival analysis (no unb adults 1st year)

D[2] <- cut(N[2])/A # density (adults/ha)

cut.p <- cut(p) # use cut function so detection prob not affected by analysis below

for (j in 3:n.surveys) { # for each subsequent breeding season...

u.unb.c[j] ~ dunif(0,10) # prior for no. undetected unbanded adults

u.unb[j] <- round(u.unb.c[j]) # convert prior to integer

n.unb[j] ~ dbin(cut.p,N.unb[j]) # sample actual number based on detection prob.

N.unb[j] <- n.unb[j]+u.unb[j] # total number of unbanded adults

N.band[j] <- sum(alive.ad[,j]) # number of banded adults from survival analysis

N[j] <- N.band[j]+N.unb[j] # total number of adults

D[j] <- cut(N[j])/A # density (adults/ha)

}

# CALCULATIONS

# back-transform vital rates and calculate lambda

log(mu.ave) <- a.f

logit(sj.ave) <- a.phi

logit(sa.ave) <- a.phi+b.age.phi

lambda <- sa.ave + 0.5*mu.ave*sj.ave

}

**Appendix S3.** Data on survival and fecundity of North Island Robins at Tawharanui Regional Park from 2007-2016.

list(

A=120 # area of forest habitat (ha)

# no. of individual females in fecundity data set

n.fem=79,

# number of fecundity observations

# select value according to number of years’ data you want to model

#n.f.obs=9, # 1 year

#n.f.obs=17, # 2 years

#n.f.obs=27, # 3 years

#n.f.obs=42, # 4 years

#n.f.obs=54, # 5 years

#n.f.obs=72, # 6 years

#n.f.obs=105, # 7 years

#n.f.obs=143, # 8 years

n.f.obs=179, # 9 years

# fecundity observations (no. young fledged by each female each breeding season)

fl=c(5,3,4,4,1,4,5,5,3,3,5,2,2,5,3,7,6,8,3,4,5,4,6,7,8,5,5,5,0,3,2,1,3,2,5,8,7,0,4,7,5,4,5,6,0,7,8,7,5,9,0,7,6,4,4,4,4,1,4,0,5,5,7,5,3,6,7,2,3,4,7,3,2,3,1,5,4,4,5,2,0,3,2,6,4,5,3,5,5,4,3,3,6,5,4,5,4,4,8,5,1,3,3,5,3,5,4,2,4,5,2,5,3,3,2,3,6,4,3,0,4,5,5,3,5,3,6,9,2,6,4,0,5,2,7,6,4,5,4,0,3,2,1,1,4,0,4,2,6,2,1,3,3,3,5,3,4,4,5,4,2,8,1,1,2,3,6,5,2,2,5,0,3,3,2,2,5,3,5),

# individual female corresponding to each fecundity observation

fem=c(1,2,3,4,5,6,7,8,9,2,4,10,5,6,8,9,1,4,11,12,13,14,15,6,8,16,17,2,2,4,18,19,20,21,12,12,13,14,6,8,16,17,4,22,23,20,12,13,6,16,17,24,25,26,27,28,29,30,4,22,23,20,12,13,6,16,31,32,24,33,25,26,34,35,36,37,38,39,40,41,42,43,44,45,46,47,28,29,48,49,4,23,20,12,13,6,16,50,31,51,52,24,33,25,26,53,35,38,39,40,45,46,54,28,29,55,48,49,4,56,57,58,59,60,61,23,20,12,13,6,16,62,50,31,51,52,63,64,65,66,24,67,26,35,37,38,54,29,48,49,68,4,69,57,58,59,70,60,61,23,20,12,71,72,6,16,50,52,65,73,74,24,67,26,75,76,77,78,79),

# year corresponding to each fecundity observation

year=c(2,2,2,2,2,2,2,2,2,3,3,3,3,3,3,3,3,4,4,4,4,4,4,4,4,4,4,5,5,5,5,5,5,5,5,5,5,5,5,5,5,5,6,6,6,6,6,6,6,6,6,6,6,6,7,7,7,7,7,7,7,7,7,7,7,7,7,7,7,7,7,7,8,8,8,8,8,8,8,8,8,8,8,8,8,8,8,8,8,8,8,8,8,8,8,8,8,8,8,8,8,8,8,8,8,9,9,9,9,9,9,9,9,9,9,9,9,9,9,9,9,9,9,9,9,9,9,9,9,9,9,9,9,9,9,9,9,9,9,9,9,9,9,10,10,10,10,10,10,10,10,10,10,10,10,10,10,10,10,10,10,10,10,10,10,10,10,10,10,10,10,10,10,10,10,10,10,10,10),

# time intervals for survival analysis (first interval from Mar-Sep 2007, annual thereafter)

int=c(NA,0.5,1,1,1,1,1,1,1,1,1),

# number of individuals in survival data set

# select value according to number of years’ data you want to model

#n.ind=57, # 1 year

#n.ind=90, # 2 years

#n.ind=146, # 3 years

#n.ind=194, # 4 years

#n.ind=247, # 5 years

#n.ind=321, # 6 years

#n.ind=438, # 7 years

#n.ind=578, # 8 years

n.ind=687, #9 years

# number of surveys in survival data set

# select value according to number of years’ data you want to model

#n.surveys=3, # 1 year

#n.surveys=4, # 2 years

#n.surveys=5, # 3 years

#n.surveys=6, # 4 years

#n.surveys=7, # 5 years

#n.surveys=8, # 6 years

#n.surveys=9, # 7 years

#n.surveys=10,# 8 years

n.surveys=11, # 9 years

# encounter histories for survival analysis

seen=structure(.Data=c(

1,1,1,1,1,1,1,1,1,1,0,

1,1,1,1,1,0,0,0,0,0,0,

1,1,1,0,0,0,0,0,0,0,0,

1,1,1,1,1,0,0,0,0,0,0,

1,0,0,0,0,0,0,0,0,0,0,

1,1,0,0,0,0,0,0,0,0,0,

1,1,1,1,1,1,1,1,1,1,1,

1,1,0,0,0,0,0,0,0,0,0,

1,1,1,1,1,1,1,1,1,1,1,

1,1,1,0,0,0,0,0,0,0,0,

1,1,1,1,1,1,1,1,0,0,0,

1,0,0,0,0,0,0,0,0,0,0,

1,1,1,0,0,0,0,0,0,0,0,

1,1,1,0,0,0,0,0,0,0,0,

1,1,1,0,0,0,0,0,0,0,0,

1,1,1,1,1,1,1,1,1,0,0,

1,0,0,0,0,0,0,0,0,0,0,

1,1,1,1,1,1,0,0,0,0,0,

1,1,1,1,1,0,0,0,0,0,0,

1,1,0,0,0,0,0,0,0,0,0,

1,1,1,1,1,1,1,1,1,0,0,

1,1,1,0,0,0,0,0,0,0,0,

1,1,1,1,1,1,1,1,1,1,1,

1,1,0,0,0,0,0,0,0,0,0,

1,1,1,1,1,0,0,0,0,0,0,

0,1,1,0,0,0,0,0,0,0,0,

0,1,0,0,0,0,0,0,0,0,0,

0,1,0,0,0,0,0,0,0,0,0,

0,1,0,0,0,0,0,0,0,0,0,

0,1,0,0,0,0,0,0,0,0,0,

0,1,0,0,0,0,0,0,0,0,0,

0,1,0,0,0,0,0,0,0,0,0,

0,1,0,0,0,0,0,0,0,0,0,

0,1,0,0,0,0,0,0,0,0,0,

0,1,0,0,0,0,0,0,0,0,0,

0,1,0,0,0,0,0,0,0,0,0,

0,1,0,0,0,0,0,0,0,0,0,

0,1,0,0,0,0,0,0,0,0,0,

0,1,1,1,0,0,0,0,0,0,0,

0,1,0,0,0,0,0,0,0,0,0,

0,1,0,0,0,0,0,0,0,0,0,

0,1,0,0,0,0,0,0,0,0,0,

0,1,0,0,0,0,0,0,0,0,0,

0,1,0,0,0,0,0,0,0,0,0,

0,1,0,0,0,0,0,0,0,0,0,

0,1,0,0,0,0,0,0,0,0,0,

0,1,0,0,0,0,0,0,0,0,0,

0,1,0,0,0,0,0,0,0,0,0,

0,1,0,0,0,0,0,0,0,0,0,

0,1,0,0,0,0,0,0,0,0,0,

0,1,0,0,0,0,0,0,0,0,0,

0,1,1,1,0,0,0,0,0,0,0,

0,1,0,0,0,0,0,0,0,0,0,

0,1,0,0,0,0,0,0,0,0,0,

0,1,0,0,0,0,0,0,0,0,0,

0,1,0,0,0,0,0,0,0,0,0,

0,1,0,0,0,0,0,0,0,0,0,

0,0,1,0,0,0,0,0,0,0,0,

0,0,1,0,0,0,0,0,0,0,0,

0,0,1,0,0,0,0,0,0,0,0,

0,0,1,1,0,0,0,0,0,0,0,

0,0,1,1,1,1,1,1,1,1,1,

0,0,1,0,0,0,0,0,0,0,0,

0,0,1,1,1,1,1,1,1,0,0,

0,0,1,1,1,0,0,0,0,0,0,

0,0,1,0,0,0,0,0,0,0,0,

0,0,1,1,0,0,0,0,0,0,0,

0,0,1,1,1,1,1,1,1,1,1,

0,0,1,0,0,0,0,0,0,0,0,

0,0,1,0,0,0,0,0,0,0,0,

0,0,1,0,0,0,0,0,0,0,0,

0,0,1,1,1,1,1,1,1,1,1,

0,0,1,0,0,0,0,0,0,0,0,

0,0,1,0,0,0,0,0,0,0,0,

0,0,1,0,0,0,0,0,0,0,0,

0,0,1,0,0,0,0,0,0,0,0,

0,0,1,1,1,1,0,0,0,0,0,

0,0,1,0,0,0,0,0,0,0,0,

0,0,1,0,0,0,0,0,0,0,0,

0,0,1,1,0,0,0,0,0,0,0,

0,0,1,0,0,0,0,0,0,0,0,

0,0,1,0,0,0,0,0,0,0,0,

0,0,1,1,1,1,0,0,0,0,0,

0,0,1,0,0,0,0,0,0,0,0,

0,0,1,0,0,0,0,0,0,0,0,

0,0,1,0,0,0,0,0,0,0,0,

0,0,1,0,0,0,0,0,0,0,0,

0,0,1,0,0,0,0,0,0,0,0,

0,0,1,0,0,0,0,0,0,0,0,

0,0,1,0,0,0,0,0,0,0,0,

0,0,0,1,0,0,0,0,0,0,0,

0,0,0,1,0,0,0,0,0,0,0,

0,0,0,1,1,1,0,0,0,0,0,

0,0,0,1,0,0,0,0,0,0,0,

0,0,0,1,1,1,1,1,1,0,0,

0,0,0,1,0,0,0,0,0,0,0,

0,0,0,1,1,0,0,0,0,0,0,

0,0,0,1,0,0,0,0,0,0,0,

0,0,0,1,0,0,0,0,0,0,0,

0,0,0,1,0,0,0,0,0,0,0,

0,0,0,1,0,0,0,0,0,0,0,

0,0,0,1,0,0,0,0,0,0,0,

0,0,0,1,0,0,0,0,0,0,0,

0,0,0,1,0,0,0,0,0,0,0,

0,0,0,1,0,0,0,0,0,0,0,

0,0,0,1,0,0,0,0,0,0,0,

0,0,0,1,0,0,0,0,0,0,0,

0,0,0,1,0,0,0,0,0,0,0,

0,0,0,1,0,0,0,0,0,0,0,

0,0,0,1,0,0,0,0,0,0,0,

0,0,0,1,1,1,1,0,0,0,0,

0,0,0,1,0,0,0,0,0,0,0,

0,0,0,1,0,0,0,0,0,0,0,

0,0,0,1,0,0,0,0,0,0,0,

0,0,0,1,0,0,0,0,0,0,0,

0,0,0,1,0,0,0,0,0,0,0,

0,0,0,1,0,0,0,0,0,0,0,

0,0,0,1,0,1,1,1,1,1,1,

0,0,0,1,1,1,1,1,1,1,0,

0,0,0,1,0,0,0,0,0,0,0,

0,0,0,1,0,0,0,0,0,0,0,

0,0,0,1,0,0,0,0,0,0,0,

0,0,0,1,0,0,0,0,0,0,0,

0,0,0,1,0,0,0,0,0,0,0,

0,0,0,1,0,0,0,0,0,0,0,

0,0,0,1,0,0,0,0,0,0,0,

0,0,0,1,1,1,1,1,1,1,1,

0,0,0,1,0,0,0,0,0,0,0,

0,0,0,1,0,0,1,0,0,0,0,

0,0,0,1,0,0,0,0,0,0,0,

0,0,0,1,0,0,0,0,0,0,0,

0,0,0,1,1,0,0,0,0,0,0,

0,0,0,1,0,0,0,0,0,0,0,

0,0,0,1,0,0,0,0,0,0,0,

0,0,0,1,0,0,0,0,0,0,0,

0,0,0,1,0,0,0,0,0,0,0,

0,0,0,1,1,0,0,0,0,0,0,

0,0,0,1,1,1,1,1,1,1,1,

0,0,0,1,0,0,0,0,0,0,0,

0,0,0,1,0,0,0,0,0,0,0,

0,0,0,1,0,0,0,0,0,0,0,

0,0,0,1,1,0,0,0,0,0,0,

0,0,0,1,0,0,0,0,0,0,0,

0,0,0,1,0,0,0,0,0,0,0,

0,0,0,1,0,0,0,0,0,0,0,

0,0,0,1,0,0,0,0,0,0,0,

0,0,0,0,1,0,0,0,0,0,0,

0,0,0,0,1,0,0,0,0,0,0,

0,0,0,0,1,0,0,0,0,0,0,

0,0,0,0,1,0,0,0,0,0,0,

0,0,0,0,1,0,0,0,0,0,0,

0,0,0,0,1,0,0,0,0,0,0,

0,0,0,0,1,0,0,0,0,0,0,

0,0,0,0,1,0,0,0,0,0,0,

0,0,0,0,1,0,0,0,0,0,0,

0,0,0,0,1,0,0,0,0,0,0,

0,0,0,0,1,0,0,0,0,0,0,

0,0,0,0,1,0,0,0,0,0,0,

0,0,0,0,1,0,0,0,0,0,0,

0,0,0,0,1,1,1,0,0,0,0,

0,0,0,0,1,0,0,0,0,0,0,

0,0,0,0,1,0,0,0,0,0,0,

0,0,0,0,1,0,0,0,0,0,0,

0,0,0,0,1,1,0,0,0,0,0,

0,0,0,0,1,1,1,1,1,1,1,

0,0,0,0,1,0,0,0,0,0,0,

0,0,0,0,1,0,0,0,0,0,0,

0,0,0,0,1,0,0,0,0,0,0,

0,0,0,0,1,0,0,0,0,0,0,

0,0,0,0,1,0,0,0,0,0,0,

0,0,0,0,1,0,0,0,0,0,0,

0,0,0,0,1,0,0,0,0,0,0,

0,0,0,0,1,0,0,0,0,0,0,

0,0,0,0,1,0,0,0,0,0,0,

0,0,0,0,1,0,0,0,0,0,0,

0,0,0,0,1,0,0,0,0,0,0,

0,0,0,0,1,0,0,0,1,1,0,

0,0,0,0,1,0,0,0,0,0,0,

0,0,0,0,1,0,0,0,0,0,0,

0,0,0,0,1,0,0,0,0,0,0,

0,0,0,0,1,0,0,0,0,0,0,

0,0,0,0,1,0,0,0,0,0,0,

0,0,0,0,1,0,0,0,0,0,0,

0,0,0,0,1,0,1,1,0,0,1,

0,0,0,0,1,0,0,0,0,0,0,

0,0,0,0,1,0,0,0,0,0,0,

0,0,0,0,1,1,1,1,0,0,0,

0,0,0,0,1,0,0,0,0,0,0,

0,0,0,0,1,0,0,0,0,0,0,

0,0,0,0,1,0,0,0,0,0,0,

0,0,0,0,1,1,1,1,1,1,1,

0,0,0,0,1,0,0,0,0,0,0,

0,0,0,0,1,0,0,0,0,0,0,

0,0,0,0,1,0,0,0,0,0,0,

0,0,0,0,0,1,1,1,1,1,1,

0,0,0,0,0,1,0,0,0,0,0,

0,0,0,0,0,1,0,0,0,0,0,

0,0,0,0,0,1,0,0,0,0,0,

0,0,0,0,0,1,0,0,0,0,0,

0,0,0,0,0,1,0,1,0,1,1,

0,0,0,0,0,1,0,0,0,0,0,

0,0,0,0,0,1,1,1,1,1,1,

0,0,0,0,0,1,0,0,0,0,0,

0,0,0,0,0,1,1,0,0,0,0,

0,0,0,0,0,1,1,0,0,0,0,

0,0,0,0,0,1,0,0,0,0,0,

0,0,0,0,0,1,1,1,1,1,0,

0,0,0,0,0,1,0,0,0,0,0,

0,0,0,0,0,1,0,0,0,0,0,

0,0,0,0,0,1,0,0,0,0,0,

0,0,0,0,0,1,0,0,0,0,0,

0,0,0,0,0,1,0,0,0,0,0,

0,0,0,0,0,1,0,0,0,0,0,

0,0,0,0,0,1,0,0,0,0,0,

0,0,0,0,0,1,0,0,0,0,0,

0,0,0,0,0,1,0,0,0,0,0,

0,0,0,0,0,1,0,0,0,0,0,

0,0,0,0,0,1,0,0,0,0,0,

0,0,0,0,0,1,0,0,0,0,0,

0,0,0,0,0,1,0,1,1,1,1,

0,0,0,0,0,1,0,0,0,0,0,

0,0,0,0,0,1,0,0,0,0,0,

0,0,0,0,0,1,1,1,1,1,1,

0,0,0,0,0,1,0,0,0,0,0,

0,0,0,0,0,1,0,0,0,0,0,

0,0,0,0,0,1,0,0,0,0,0,

0,0,0,0,0,1,1,1,1,0,0,

0,0,0,0,0,1,0,0,0,0,0,

0,0,0,0,0,1,1,1,1,1,0,

0,0,0,0,0,1,0,0,0,0,0,

0,0,0,0,0,1,0,0,1,0,0,

0,0,0,0,0,1,1,0,0,0,0,

0,0,0,0,0,1,0,0,0,0,0,

0,0,0,0,0,1,0,1,1,1,1,

0,0,0,0,0,1,0,0,0,0,0,

0,0,0,0,0,1,0,0,0,0,0,

0,0,0,0,0,1,0,0,0,0,0,

0,0,0,0,0,1,0,0,0,0,0,

0,0,0,0,0,1,0,0,0,0,0,

0,0,0,0,0,1,0,0,1,0,0,

0,0,0,0,0,1,0,0,0,0,0,

0,0,0,0,0,1,0,0,0,0,0,

0,0,0,0,0,1,0,1,1,1,1,

0,0,0,0,0,1,0,0,0,0,0,

0,0,0,0,0,1,0,0,0,0,0,

0,0,0,0,0,1,0,0,0,0,0,

0,0,0,0,0,1,0,0,0,0,0,

0,0,0,0,0,0,1,0,0,0,0,

0,0,0,0,0,0,1,1,0,0,0,

0,0,0,0,0,0,1,1,1,1,0,

0,0,0,0,0,0,1,1,0,0,0,

0,0,0,0,0,0,1,0,0,0,0,

0,0,0,0,0,0,1,0,0,0,0,

0,0,0,0,0,0,1,1,0,0,0,

0,0,0,0,0,0,1,1,1,1,0,

0,0,0,0,0,0,1,0,0,0,0,

0,0,0,0,0,0,1,0,0,0,0,

0,0,0,0,0,0,1,0,0,0,0,

0,0,0,0,0,0,1,0,0,0,0,

0,0,0,0,0,0,1,0,1,1,1,

0,0,0,0,0,0,1,0,0,0,0,

0,0,0,0,0,0,1,1,1,1,1,

0,0,0,0,0,0,1,1,1,1,1,

0,0,0,0,0,0,1,0,0,0,0,

0,0,0,0,0,0,1,0,0,0,0,

0,0,0,0,0,0,1,0,0,0,0,

0,0,0,0,0,0,1,0,0,0,0,

0,0,0,0,0,0,1,0,0,0,0,

0,0,0,0,0,0,1,1,1,1,0,

0,0,0,0,0,0,1,1,0,0,0,

0,0,0,0,0,0,1,1,1,0,0,

0,0,0,0,0,0,1,1,1,1,1,

0,0,0,0,0,0,1,0,0,0,0,

0,0,0,0,0,0,1,1,1,0,0,

0,0,0,0,0,0,1,0,0,0,0,

0,0,0,0,0,0,1,0,0,0,0,

0,0,0,0,0,0,1,0,0,0,0,

0,0,0,0,0,0,1,0,0,0,0,

0,0,0,0,0,0,1,1,1,1,0,

0,0,0,0,0,0,1,0,0,0,0,

0,0,0,0,0,0,1,1,0,0,0,

0,0,0,0,0,0,1,0,0,0,0,

0,0,0,0,0,0,1,0,0,0,0,

0,0,0,0,0,0,1,1,0,0,0,

0,0,0,0,0,0,1,0,0,0,0,

0,0,0,0,0,0,1,0,0,0,0,

0,0,0,0,0,0,1,0,0,0,0,

0,0,0,0,0,0,1,0,0,0,0,

0,0,0,0,0,0,1,0,0,0,0,

0,0,0,0,0,0,1,0,0,0,0,

0,0,0,0,0,0,1,0,0,0,0,

0,0,0,0,0,0,1,1,0,0,0,

0,0,0,0,0,0,1,1,1,1,0,

0,0,0,0,0,0,1,0,0,0,0,

0,0,0,0,0,0,1,1,0,0,0,

0,0,0,0,0,0,1,1,1,1,1,

0,0,0,0,0,0,1,0,0,0,0,

0,0,0,0,0,0,1,0,0,0,0,

0,0,0,0,0,0,1,0,0,0,0,

0,0,0,0,0,0,1,1,1,1,0,

0,0,0,0,0,0,1,1,1,0,0,

0,0,0,0,0,0,1,0,0,0,0,

0,0,0,0,0,0,1,0,0,0,0,

0,0,0,0,0,0,1,1,0,0,0,

0,0,0,0,0,0,1,1,1,1,1,

0,0,0,0,0,0,1,0,1,0,0,

0,0,0,0,0,0,1,0,0,0,0,

0,0,0,0,0,0,1,0,0,0,0,

0,0,0,0,0,0,1,1,0,0,0,

0,0,0,0,0,0,1,1,1,0,0,

0,0,0,0,0,0,1,0,0,0,0,

0,0,0,0,0,0,1,1,0,0,0,

0,0,0,0,0,0,1,0,0,0,0,

0,0,0,0,0,0,1,0,0,0,0,

0,0,0,0,0,0,1,0,0,0,0,

0,0,0,0,0,0,1,0,0,0,0,

0,0,0,0,0,0,1,0,0,0,1,

0,0,0,0,0,0,1,0,0,0,0,

0,0,0,0,0,0,1,0,0,0,0,

0,0,0,0,0,0,1,1,1,1,1,

0,0,0,0,0,0,1,0,0,0,0,

0,0,0,0,0,0,0,1,0,0,0,

0,0,0,0,0,0,0,1,1,0,0,

0,0,0,0,0,0,0,1,0,0,0,

0,0,0,0,0,0,0,1,0,0,0,

0,0,0,0,0,0,0,1,0,0,0,

0,0,0,0,0,0,0,1,0,0,0,

0,0,0,0,0,0,0,1,1,1,1,

0,0,0,0,0,0,0,1,0,0,0,

0,0,0,0,0,0,0,1,1,1,1,

0,0,0,0,0,0,0,1,1,0,0,

0,0,0,0,0,0,0,1,0,0,0,

0,0,0,0,0,0,0,1,0,0,0,

0,0,0,0,0,0,0,1,0,0,0,

0,0,0,0,0,0,0,1,0,0,0,

0,0,0,0,0,0,0,1,0,0,0,

0,0,0,0,0,0,0,1,0,0,0,

0,0,0,0,0,0,0,1,0,0,0,

0,0,0,0,0,0,0,1,1,0,0,

0,0,0,0,0,0,0,1,0,0,0,

0,0,0,0,0,0,0,1,1,1,1,

0,0,0,0,0,0,0,1,1,1,1,

0,0,0,0,0,0,0,1,0,0,0,

0,0,0,0,0,0,0,1,0,0,0,

0,0,0,0,0,0,0,1,1,1,0,

0,0,0,0,0,0,0,1,0,0,0,

0,0,0,0,0,0,0,1,0,0,0,

0,0,0,0,0,0,0,1,0,0,0,

0,0,0,0,0,0,0,1,0,0,0,

0,0,0,0,0,0,0,1,0,0,0,

0,0,0,0,0,0,0,1,0,0,0,

0,0,0,0,0,0,0,1,0,0,0,

0,0,0,0,0,0,0,1,0,0,0,

0,0,0,0,0,0,0,1,0,0,0,

0,0,0,0,0,0,0,1,0,0,0,

0,0,0,0,0,0,0,1,0,0,0,

0,0,0,0,0,0,0,1,0,0,0,

0,0,0,0,0,0,0,1,0,0,0,

0,0,0,0,0,0,0,1,0,0,0,

0,0,0,0,0,0,0,1,0,0,0,

0,0,0,0,0,0,0,1,0,0,0,

0,0,0,0,0,0,0,1,0,0,0,

0,0,0,0,0,0,0,1,1,1,0,

0,0,0,0,0,0,0,1,0,0,0,

0,0,0,0,0,0,0,1,1,1,1,

0,0,0,0,0,0,0,1,0,0,0,

0,0,0,0,0,0,0,1,0,0,0,

0,0,0,0,0,0,0,1,0,0,0,

0,0,0,0,0,0,0,1,0,0,0,

0,0,0,0,0,0,0,1,1,1,0,

0,0,0,0,0,0,0,1,0,0,0,

0,0,0,0,0,0,0,1,0,0,0,

0,0,0,0,0,0,0,1,0,0,0,

0,0,0,0,0,0,0,1,0,0,0,

0,0,0,0,0,0,0,1,0,0,1,

0,0,0,0,0,0,0,1,0,0,0,

0,0,0,0,0,0,0,1,0,0,0,

0,0,0,0,0,0,0,1,0,0,0,

0,0,0,0,0,0,0,1,1,1,1,

0,0,0,0,0,0,0,1,0,0,0,

0,0,0,0,0,0,0,1,0,0,0,

0,0,0,0,0,0,0,1,0,0,0,

0,0,0,0,0,0,0,1,0,0,0,

0,0,0,0,0,0,0,1,0,0,0,

0,0,0,0,0,0,0,1,0,0,0,

0,0,0,0,0,0,0,1,0,0,0,

0,0,0,0,0,0,0,1,0,0,0,

0,0,0,0,0,0,0,1,0,0,0,

0,0,0,0,0,0,0,1,0,0,0,

0,0,0,0,0,0,0,1,0,0,0,

0,0,0,0,0,0,0,1,0,0,0,

0,0,0,0,0,0,0,1,0,0,0,

0,0,0,0,0,0,0,1,0,0,0,

0,0,0,0,0,0,0,1,1,1,0,

0,0,0,0,0,0,0,1,0,0,0,

0,0,0,0,0,0,0,1,0,0,0,

0,0,0,0,0,0,0,1,0,0,0,

0,0,0,0,0,0,0,1,0,0,0,

0,0,0,0,0,0,0,1,0,0,0,

0,0,0,0,0,0,0,1,0,0,0,

0,0,0,0,0,0,0,1,0,0,0,

0,0,0,0,0,0,0,1,0,0,0,

0,0,0,0,0,0,0,1,1,0,0,

0,0,0,0,0,0,0,1,0,0,0,

0,0,0,0,0,0,0,1,0,0,0,

0,0,0,0,0,0,0,1,0,0,0,

0,0,0,0,0,0,0,1,1,1,0,

0,0,0,0,0,0,0,1,0,0,0,

0,0,0,0,0,0,0,1,0,0,0,

0,0,0,0,0,0,0,1,0,0,0,

0,0,0,0,0,0,0,1,0,0,0,

0,0,0,0,0,0,0,1,0,0,0,

0,0,0,0,0,0,0,1,0,0,0,

0,0,0,0,0,0,0,1,0,0,0,

0,0,0,0,0,0,0,1,1,0,0,

0,0,0,0,0,0,0,1,0,0,0,

0,0,0,0,0,0,0,1,0,0,0,

0,0,0,0,0,0,0,1,0,0,0,

0,0,0,0,0,0,0,1,0,0,0,

0,0,0,0,0,0,0,1,0,0,0,

0,0,0,0,0,0,0,1,0,0,0,

0,0,0,0,0,0,0,1,0,0,0,

0,0,0,0,0,0,0,1,0,0,0,

0,0,0,0,0,0,0,1,0,0,0,

0,0,0,0,0,0,0,1,0,0,0,

0,0,0,0,0,0,0,1,0,0,0,

0,0,0,0,0,0,0,1,0,0,0,

0,0,0,0,0,0,0,1,0,0,0,

0,0,0,0,0,0,0,1,0,0,0,

0,0,0,0,0,0,0,1,0,0,0,

0,0,0,0,0,0,0,1,0,0,0,

0,0,0,0,0,0,0,1,0,0,0,

0,0,0,0,0,0,0,1,0,0,0,

0,0,0,0,0,0,0,1,0,0,0,

0,0,0,0,0,0,0,1,0,1,0,

0,0,0,0,0,0,0,1,0,0,0,

0,0,0,0,0,0,0,1,0,0,0,

0,0,0,0,0,0,0,1,0,0,0,

0,0,0,0,0,0,0,0,1,0,0,

0,0,0,0,0,0,0,0,1,0,0,

0,0,0,0,0,0,0,0,1,0,0,

0,0,0,0,0,0,0,0,1,0,0,

0,0,0,0,0,0,0,0,1,0,0,

0,0,0,0,0,0,0,0,1,0,0,

0,0,0,0,0,0,0,0,1,0,0,

0,0,0,0,0,0,0,0,1,1,1,

0,0,0,0,0,0,0,0,1,0,0,

0,0,0,0,0,0,0,0,1,0,0,

0,0,0,0,0,0,0,0,1,0,0,

0,0,0,0,0,0,0,0,1,0,0,

0,0,0,0,0,0,0,0,1,1,1,

0,0,0,0,0,0,0,0,1,0,0,

0,0,0,0,0,0,0,0,1,0,0,

0,0,0,0,0,0,0,0,1,0,0,

0,0,0,0,0,0,0,0,1,0,0,

0,0,0,0,0,0,0,0,1,0,0,

0,0,0,0,0,0,0,0,1,0,0,

0,0,0,0,0,0,0,0,1,1,1,

0,0,0,0,0,0,0,0,1,0,0,

0,0,0,0,0,0,0,0,1,0,0,

0,0,0,0,0,0,0,0,1,0,0,

0,0,0,0,0,0,0,0,1,0,0,

0,0,0,0,0,0,0,0,1,0,0,

0,0,0,0,0,0,0,0,1,0,0,

0,0,0,0,0,0,0,0,1,0,0,

0,0,0,0,0,0,0,0,1,0,0,

0,0,0,0,0,0,0,0,1,0,0,

0,0,0,0,0,0,0,0,1,0,0,

0,0,0,0,0,0,0,0,1,0,0,

0,0,0,0,0,0,0,0,1,0,0,

0,0,0,0,0,0,0,0,1,0,0,

0,0,0,0,0,0,0,0,1,0,0,

0,0,0,0,0,0,0,0,1,0,0,

0,0,0,0,0,0,0,0,1,0,0,

0,0,0,0,0,0,0,0,1,0,0,

0,0,0,0,0,0,0,0,1,0,0,

0,0,0,0,0,0,0,0,1,0,0,

0,0,0,0,0,0,0,0,1,0,0,

0,0,0,0,0,0,0,0,1,0,0,

0,0,0,0,0,0,0,0,1,0,0,

0,0,0,0,0,0,0,0,1,1,1,

0,0,0,0,0,0,0,0,1,0,0,

0,0,0,0,0,0,0,0,1,0,0,

0,0,0,0,0,0,0,0,1,0,0,

0,0,0,0,0,0,0,0,1,0,0,

0,0,0,0,0,0,0,0,1,0,0,

0,0,0,0,0,0,0,0,1,0,0,

0,0,0,0,0,0,0,0,1,0,0,

0,0,0,0,0,0,0,0,1,0,0,

0,0,0,0,0,0,0,0,1,0,0,

0,0,0,0,0,0,0,0,1,0,0,

0,0,0,0,0,0,0,0,1,0,0,

0,0,0,0,0,0,0,0,1,0,0,

0,0,0,0,0,0,0,0,1,0,0,

0,0,0,0,0,0,0,0,1,1,1,

0,0,0,0,0,0,0,0,1,0,0,

0,0,0,0,0,0,0,0,1,0,0,

0,0,0,0,0,0,0,0,1,0,0,

0,0,0,0,0,0,0,0,1,1,1,

0,0,0,0,0,0,0,0,1,0,0,

0,0,0,0,0,0,0,0,1,0,0,

0,0,0,0,0,0,0,0,1,0,0,

0,0,0,0,0,0,0,0,1,0,0,

0,0,0,0,0,0,0,0,1,0,0,

0,0,0,0,0,0,0,0,1,0,0,

0,0,0,0,0,0,0,0,1,1,0,

0,0,0,0,0,0,0,0,1,0,0,

0,0,0,0,0,0,0,0,1,0,0,

0,0,0,0,0,0,0,0,1,0,0,

0,0,0,0,0,0,0,0,1,1,1,

0,0,0,0,0,0,0,0,1,0,0,

0,0,0,0,0,0,0,0,1,0,0,

0,0,0,0,0,0,0,0,1,1,1,

0,0,0,0,0,0,0,0,1,0,0,

0,0,0,0,0,0,0,0,1,0,0,

0,0,0,0,0,0,0,0,1,0,0,

0,0,0,0,0,0,0,0,1,0,0,

0,0,0,0,0,0,0,0,1,0,0,

0,0,0,0,0,0,0,0,1,0,0,

0,0,0,0,0,0,0,0,1,0,0,

0,0,0,0,0,0,0,0,1,0,0,

0,0,0,0,0,0,0,0,1,0,0,

0,0,0,0,0,0,0,0,1,0,0,

0,0,0,0,0,0,0,0,1,0,0,

0,0,0,0,0,0,0,0,1,1,0,

0,0,0,0,0,0,0,0,1,0,0,

0,0,0,0,0,0,0,0,1,0,0,

0,0,0,0,0,0,0,0,1,1,1,

0,0,0,0,0,0,0,0,1,1,0,

0,0,0,0,0,0,0,0,1,0,0,

0,0,0,0,0,0,0,0,1,0,1,

0,0,0,0,0,0,0,0,1,0,0,

0,0,0,0,0,0,0,0,1,0,0,

0,0,0,0,0,0,0,0,1,0,0,

0,0,0,0,0,0,0,0,1,0,0,

0,0,0,0,0,0,0,0,1,0,0,

0,0,0,0,0,0,0,0,1,0,0,

0,0,0,0,0,0,0,0,1,0,0,

0,0,0,0,0,0,0,0,1,0,0,

0,0,0,0,0,0,0,0,1,0,0,

0,0,0,0,0,0,0,0,1,0,0,

0,0,0,0,0,0,0,0,1,0,0,

0,0,0,0,0,0,0,0,1,0,0,

0,0,0,0,0,0,0,0,1,0,0,

0,0,0,0,0,0,0,0,1,0,0,

0,0,0,0,0,0,0,0,1,0,0,

0,0,0,0,0,0,0,0,1,0,0,

0,0,0,0,0,0,0,0,1,0,0,

0,0,0,0,0,0,0,0,1,0,0,

0,0,0,0,0,0,0,0,1,0,0,

0,0,0,0,0,0,0,0,1,0,0,

0,0,0,0,0,0,0,0,1,0,0,

0,0,0,0,0,0,0,0,1,1,0,

0,0,0,0,0,0,0,0,1,0,0,

0,0,0,0,0,0,0,0,1,0,0,

0,0,0,0,0,0,0,0,1,0,0,

0,0,0,0,0,0,0,0,1,1,1,

0,0,0,0,0,0,0,0,1,0,0,

0,0,0,0,0,0,0,0,1,1,1,

0,0,0,0,0,0,0,0,1,0,0,

0,0,0,0,0,0,0,0,1,0,0,

0,0,0,0,0,0,0,0,1,0,0,

0,0,0,0,0,0,0,0,1,0,0,

0,0,0,0,0,0,0,0,1,0,0,

0,0,0,0,0,0,0,0,1,0,0,

0,0,0,0,0,0,0,0,1,0,0,

0,0,0,0,0,0,0,0,1,1,1,

0,0,0,0,0,0,0,0,1,0,0,

0,0,0,0,0,0,0,0,1,0,0,

0,0,0,0,0,0,0,0,1,0,0,

0,0,0,0,0,0,0,0,1,0,0,

0,0,0,0,0,0,0,0,1,0,0,

0,0,0,0,0,0,0,0,1,0,0,

0,0,0,0,0,0,0,0,1,0,0,

0,0,0,0,0,0,0,0,1,0,0,

0,0,0,0,0,0,0,0,1,0,0,

0,0,0,0,0,0,0,0,1,0,0,

0,0,0,0,0,0,0,0,1,0,0,

0,0,0,0,0,0,0,0,0,1,0,

0,0,0,0,0,0,0,0,0,1,0,

0,0,0,0,0,0,0,0,0,1,0,

0,0,0,0,0,0,0,0,0,1,0,

0,0,0,0,0,0,0,0,0,1,0,

0,0,0,0,0,0,0,0,0,1,0,

0,0,0,0,0,0,0,0,0,1,0,

0,0,0,0,0,0,0,0,0,1,0,

0,0,0,0,0,0,0,0,0,1,0,

0,0,0,0,0,0,0,0,0,1,1,

0,0,0,0,0,0,0,0,0,1,0,

0,0,0,0,0,0,0,0,0,1,0,

0,0,0,0,0,0,0,0,0,1,0,

0,0,0,0,0,0,0,0,0,1,1,

0,0,0,0,0,0,0,0,0,1,1,

0,0,0,0,0,0,0,0,0,1,0,

0,0,0,0,0,0,0,0,0,1,0,

0,0,0,0,0,0,0,0,0,1,0,

0,0,0,0,0,0,0,0,0,1,0,

0,0,0,0,0,0,0,0,0,1,0,

0,0,0,0,0,0,0,0,0,1,0,

0,0,0,0,0,0,0,0,0,1,0,

0,0,0,0,0,0,0,0,0,1,1,

0,0,0,0,0,0,0,0,0,1,0,

0,0,0,0,0,0,0,0,0,1,1,

0,0,0,0,0,0,0,0,0,1,0,

0,0,0,0,0,0,0,0,0,1,1,

0,0,0,0,0,0,0,0,0,1,0,

0,0,0,0,0,0,0,0,0,1,0,

0,0,0,0,0,0,0,0,0,1,0,

0,0,0,0,0,0,0,0,0,1,0,

0,0,0,0,0,0,0,0,0,1,0,

0,0,0,0,0,0,0,0,0,1,0,

0,0,0,0,0,0,0,0,0,1,0,

0,0,0,0,0,0,0,0,0,1,1,

0,0,0,0,0,0,0,0,0,1,0,

0,0,0,0,0,0,0,0,0,1,0,

0,0,0,0,0,0,0,0,0,1,0,

0,0,0,0,0,0,0,0,0,1,0,

0,0,0,0,0,0,0,0,0,1,0,

0,0,0,0,0,0,0,0,0,1,0,

0,0,0,0,0,0,0,0,0,1,0,

0,0,0,0,0,0,0,0,0,1,0,

0,0,0,0,0,0,0,0,0,1,0,

0,0,0,0,0,0,0,0,0,1,0,

0,0,0,0,0,0,0,0,0,1,0,

0,0,0,0,0,0,0,0,0,1,1,

0,0,0,0,0,0,0,0,0,1,1,

0,0,0,0,0,0,0,0,0,1,0,

0,0,0,0,0,0,0,0,0,1,0,

0,0,0,0,0,0,0,0,0,1,0,

0,0,0,0,0,0,0,0,0,1,0,

0,0,0,0,0,0,0,0,0,1,0,

0,0,0,0,0,0,0,0,0,1,0,

0,0,0,0,0,0,0,0,0,1,0,

0,0,0,0,0,0,0,0,0,1,0,

0,0,0,0,0,0,0,0,0,1,0,

0,0,0,0,0,0,0,0,0,1,0,

0,0,0,0,0,0,0,0,0,1,1,

0,0,0,0,0,0,0,0,0,1,0,

0,0,0,0,0,0,0,0,0,1,0,

0,0,0,0,0,0,0,0,0,1,1,

0,0,0,0,0,0,0,0,0,1,0,

0,0,0,0,0,0,0,0,0,1,0,

0,0,0,0,0,0,0,0,0,1,1,

0,0,0,0,0,0,0,0,0,1,0,

0,0,0,0,0,0,0,0,0,1,0,

0,0,0,0,0,0,0,0,0,1,0,

0,0,0,0,0,0,0,0,0,1,0,

0,0,0,0,0,0,0,0,0,1,1,

0,0,0,0,0,0,0,0,0,1,0,

0,0,0,0,0,0,0,0,0,1,0,

0,0,0,0,0,0,0,0,0,1,0,

0,0,0,0,0,0,0,0,0,1,0,

0,0,0,0,0,0,0,0,0,1,0,

0,0,0,0,0,0,0,0,0,1,0,

0,0,0,0,0,0,0,0,0,1,0,

0,0,0,0,0,0,0,0,0,1,0,

0,0,0,0,0,0,0,0,0,1,0,

0,0,0,0,0,0,0,0,0,1,0,

0,0,0,0,0,0,0,0,0,1,0,

0,0,0,0,0,0,0,0,0,1,1,

0,0,0,0,0,0,0,0,0,1,0,

0,0,0,0,0,0,0,0,0,1,0,

0,0,0,0,0,0,0,0,0,1,0,

0,0,0,0,0,0,0,0,0,1,1,

0,0,0,0,0,0,0,0,0,1,0,

0,0,0,0,0,0,0,0,0,1,0,

0,0,0,0,0,0,0,0,0,1,0,

0,0,0,0,0,0,0,0,0,1,0,

0,0,0,0,0,0,0,0,0,1,0,

0,0,0,0,0,0,0,0,0,1,0,

0,0,0,0,0,0,0,0,0,1,0,

0,0,0,0,0,0,0,0,0,1,0,

0,0,0,0,0,0,0,0,0,1,0,

0,0,0,0,0,0,0,0,0,1,0,

0,0,0,0,0,0,0,0,0,1,0,

0,0,0,0,0,0,0,0,0,1,0,

0,0,0,0,0,0,0,0,0,1,0,

0,0,0,0,0,0,0,0,0,1,0,

0,0,0,0,0,0,0,0,0,1,0,

0,0,0,0,0,0,0,0,0,1,0,

0,0,0,0,0,0,0,0,0,1,0,

0,0,0,0,0,0,0,0,0,1,0,

0,0,0,0,0,0,0,0,0,1,0,

0,0,0,0,0,0,0,0,0,1,0,

0,0,0,0,0,0,0,0,0,1,0,

0,0,0,0,0,0,0,0,0,1,0,

0,0,0,0,0,0,0,0,0,1,0

),.Dim = c(687, 11)),

# survey when each individual was first encountered

first=c(1,1,1,1,1,1,1,1,1,1,1,1,1,1,1,1,1,1,1,1,1,1,1,1,1,2,2,2,2,2,2,2,2,2,2,2,2,2,2,2,2,2,2,2,2,2,2,2,2,2,2,2,2,2,2,2,2,3,3,3,3,3,3,3,3,3,3,3,3,3,3,3,3,3,3,3,3,3,3,3,3,3,3,3,3,3,3,3,3,3,4,4,4,4,4,4,4,4,4,4,4,4,4,4,4,4,4,4,4,4,4,4,4,4,4,4,4,4,4,4,4,4,4,4,4,4,4,4,4,4,4,4,4,4,4,4,4,4,4,4,4,4,4,4,4,4,5,5,5,5,5,5,5,5,5,5,5,5,5,5,5,5,5,5,5,5,5,5,5,5,5,5,5,5,5,5,5,5,5,5,5,5,5,5,5,5,5,5,5,5,5,5,5,5,6,6,6,6,6,6,6,6,6,6,6,6,6,6,6,6,6,6,6,6,6,6,6,6,6,6,6,6,6,6,6,6,6,6,6,6,6,6,6,6,6,6,6,6,6,6,6,6,6,6,6,6,6,7,7,7,7,7,7,7,7,7,7,7,7,7,7,7,7,7,7,7,7,7,7,7,7,7,7,7,7,7,7,7,7,7,7,7,7,7,7,7,7,7,7,7,7,7,7,7,7,7,7,7,7,7,7,7,7,7,7,7,7,7,7,7,7,7,7,7,7,7,7,7,7,7,7,8,8,8,8,8,8,8,8,8,8,8,8,8,8,8,8,8,8,8,8,8,8,8,8,8,8,8,8,8,8,8,8,8,8,8,8,8,8,8,8,8,8,8,8,8,8,8,8,8,8,8,8,8,8,8,8,8,8,8,8,8,8,8,8,8,8,8,8,8,8,8,8,8,8,8,8,8,8,8,8,8,8,8,8,8,8,8,8,8,8,8,8,8,8,8,8,8,8,8,8,8,8,8,8,8,8,8,8,8,8,8,8,8,8,8,8,8,9,9,9,9,9,9,9,9,9,9,9,9,9,9,9,9,9,9,9,9,9,9,9,9,9,9,9,9,9,9,9,9,9,9,9,9,9,9,9,9,9,9,9,9,9,9,9,9,9,9,9,9,9,9,9,9,9,9,9,9,9,9,9,9,9,9,9,9,9,9,9,9,9,9,9,9,9,9,9,9,9,9,9,9,9,9,9,9,9,9,9,9,9,9,9,9,9,9,9,9,9,9,9,9,9,9,9,9,9,9,9,9,9,9,9,9,9,9,9,9,9,9,9,9,9,9,9,9,9,9,9,9,9,9,9,9,9,9,9,9,10,10,10,10,10,10,10,10,10,10,10,10,10,10,10,10,10,10,10,10,10,10,10,10,10,10,10,10,10,10,10,10,10,10,10,10,10,10,10,10,10,10,10,10,10,10,10,10,10,10,10,10,10,10,10,10,10,10,10,10,10,10,10,10,10,10,10,10,10,10,10,10,10,10,10,10,10,10,10,10,10,10,10,10,10,10,10,10,10,10,10,10,10,10,10,10,10,10,10,10,10,10,10,10,10,10,10,10,10),

# whether each individual in matrix was translocated (0=No, 1=Yes)

trans=c(1,1,1,1,1,1,1,1,1,1,1,1,1,1,1,1,1,1,1,1,1,1,1,1,1,0,0,0,0,0,0,0,0,0,0,0,0,0,0,0,0,0,0,0,0,0,0,0,0,0,0,0,0,0,0,0,0,0,0,0,0,0,0,0,0,0,0,0,0,0,0,0,0,0,0,0,0,0,0,0,0,0,0,0,0,0,0,0,0,0,0,0,0,0,0,0,0,0,0,0,0,0,0,0,0,0,0,0,0,0,0,0,0,0,0,0,0,0,0,0,0,0,0,0,0,0,0,0,0,0,0,0,0,0,0,0,0,0,0,0,0,0,0,0,0,0,0,0,0,0,0,0,0,0,0,0,0,0,0,0,0,0,0,0,0,0,0,0,0,0,0,0,0,0,0,0,0,0,0,0,0,0,0,0,0,0,0,0,0,0,0,0,0,0,0,0,0,0,0,0,0,0,0,0,0,0,0,0,0,0,0,0,0,0,0,0,0,0,0,0,0,0,0,0,0,0,0,0,0,0,0,0,0,0,0,0,0,0,0,0,0,0,0,0,0,0,0,0,0,0,0,0,0,0,0,0,0,0,0,0,0,0,0,0,0,0,0,0,0,0,0,0,0,0,0,0,0,0,0,0,0,0,0,0,0,0,0,0,0,0,0,0,0,0,0,0,0,0,0,0,0,0,0,0,0,0,0,0,0,0,0,0,0,0,0,0,0,0,0,0,0,0,0,0,0,0,0,0,0,0,0,0,0,0,0,0,0,0,0,0,0,0,0,0,0,0,0,0,0,0,0,0,0,0,0,0,0,0,0,0,0,0,0,0,0,0,0,0,0,0,0,0,0,0,0,0,0,0,0,0,0,0,0,0,0,0,0,0,0,0,0,0,0,0,0,0,0,0,0,0,0,0,0,0,0,0,0,0,0,0,0,0,0,0,0,0,0,0,0,0,0,0,0,0,0,0,0,0,0,0,0,0,0,0,0,0,0,0,0,0,0,0,0,0,0,0,0,0,0,0,0,0,0,0,0,0,0,0,0,0,0,0,0,0,0,0,0,0,0,0,0,0,0,0,0,0,0,0,0,0,0,0,0,0,0,0,0,0,0,0,0,0,0,0,0,0,0,0,0,0,0,0,0,0,0,0,0,0,0,0,0,0,0,0,0,0,0,0,0,0,0,0,0,0,0,0,0,0,0,0,0,0,0,0,0,0,0,0,0,0,0,0,0,0,0,0,0,0,0,0,0,0,0,0,0,0,0,0,0,0,0,0,0,0,0,0,0,0,0,0,0,0,0,0,0,0,0,0,0,0,0,0,0,0,0,0,0,0,0,0,0,0,0,0,0,0,0,0,0,0,0,0,0,0,0,0,0,0,0,0,0,0,0,0,0,0,0,0,0,0,0,0,0,0,0,0,0,0,0,0,0,0,0,0,0,0,0,0,0,0,0,0,0,0,0,0,0,0,0,0,0,0,0,0,0,0,0,0,0,0,0,0,0,0,0,0,0,0,0,0,0,0,0,0,0,0,0,0,0,0,0,0,0,0,0,0,0),

# whether each individual in encounter matrix was banded as a fledgling (0=no, 1=yes)

# this avoids bias due to subsequent survival probability being higher if banded at this stage

bfl=c(0,0,0,0,0,0,0,0,0,0,0,0,0,0,0,0,0,0,0,0,0,0,0,0,0,0,0,0,0,0,0,0,0,0,0,0,0,0,0,0,0,0,0,0,0,0,0,0,0,0,0,0,0,0,0,0,0,0,0,0,0,0,0,0,0,0,0,0,0,0,0,0,0,0,0,0,0,0,0,0,0,0,0,0,0,0,0,0,0,0,0,0,0,0,0,0,0,0,0,0,0,0,0,0,0,0,0,0,0,0,0,0,0,0,0,0,0,0,0,0,0,0,0,0,0,0,0,0,0,0,0,0,0,0,0,0,0,0,0,0,0,0,0,0,0,0,0,0,0,0,0,0,0,0,0,0,0,0,0,0,0,0,0,0,0,0,0,0,0,0,0,0,0,0,0,0,0,0,0,0,0,0,0,0,0,0,0,0,0,0,0,0,0,0,1,0,0,0,0,0,0,0,0,0,0,0,0,0,0,0,0,0,0,0,0,0,0,0,0,0,0,0,0,0,0,0,0,0,0,0,0,0,0,0,0,0,0,0,0,0,0,0,0,0,0,0,0,0,0,0,0,0,0,0,0,0,0,0,0,0,0,0,0,0,0,0,0,0,0,0,0,0,0,0,0,0,0,0,0,0,0,0,0,0,0,0,0,0,0,0,0,0,0,0,0,0,0,0,0,0,0,0,0,0,0,0,0,0,0,0,0,1,0,0,0,0,0,0,0,0,0,0,0,0,0,0,0,0,0,0,0,0,0,0,0,0,0,0,0,0,0,0,0,0,0,0,0,0,0,0,0,0,0,0,0,0,0,0,0,0,0,0,0,0,0,0,0,0,0,0,0,0,0,0,0,0,0,0,0,0,0,0,0,0,0,0,0,0,0,0,0,0,0,0,0,0,0,0,0,0,0,0,0,0,0,0,0,0,0,0,0,0,0,0,0,0,0,0,0,0,0,0,0,0,0,0,0,0,0,0,0,0,0,0,0,0,0,0,0,0,0,0,0,0,0,0,0,0,0,0,0,0,0,0,1,0,0,1,0,0,0,0,0,0,0,0,0,0,0,0,0,0,0,0,0,0,0,0,0,0,0,0,0,0,0,0,0,0,0,0,0,0,0,0,0,0,0,0,0,0,0,0,0,0,0,0,0,0,0,0,0,0,0,0,0,0,0,0,0,0,0,0,0,0,0,0,0,0,0,0,0,0,0,0,0,0,0,0,0,0,0,0,0,0,0,0,0,0,0,0,0,0,0,0,0,0,0,0,0,0,0,0,0,0,0,0,0,0,0,0,0,0,0,0,0,0,0,0,0,0,0,0,0,0,0,0,0,0,0,0,0,0,0,0,0,0,0,0,0,0,0,0,0,0,0,0,0,0,0,0,0,0,0,0,0,0,0,0,0,0,0,0,0,0,0,0,0,0,0,0,0,0,0,0,0,0,0,0,0,0,0,0,0,0,0,0,0,0,0,0,0,0,0,0,0,0,0,0,0,0,0,0,0,0,0,0,0,0,0,0,0,0,0,0,0,0,0,0,0,0,0,0,0,0,0,0,0),

# sexes of individuals in encounter matrix (1=male, 0=female, NA=unknown)

sex=c(1,1,0,0,1,0,0,1,1,1,1,0,1,0,1,1,1,1,1,1,1,0,0,0,0,0,NA,NA,NA,NA,NA,NA,NA,NA,NA,NA,NA,NA,1,NA,NA,NA,NA,NA,NA,NA,NA,NA,NA,NA,NA,1,NA,NA,NA,NA,NA,NA,NA,NA,0,0,NA,0,0,NA,0,0,NA,NA,NA,1,NA,NA,NA,NA,0,NA,NA,1,NA,NA,1,NA,NA,NA,NA,NA,NA,NA,NA,NA,1,NA,1,NA,0,NA,NA,NA,NA,NA,NA,NA,NA,NA,NA,NA,NA,NA,0,NA,NA,NA,NA,NA,NA,0,1,NA,NA,NA,NA,NA,NA,NA,1,NA,1,NA,NA,0,NA,NA,NA,NA,1,0,NA,NA,NA,0,NA,NA,NA,NA,NA,NA,NA,NA,NA,NA,NA,NA,NA,NA,NA,NA,NA,1,NA,NA,NA,1,0,NA,NA,NA,NA,NA,NA,NA,NA,NA,NA,NA,0,NA,NA,NA,NA,NA,NA,0,NA,NA,0,NA,NA,NA,0,NA,NA,NA,1,NA,NA,NA,NA,1,NA,1,NA,0,1,NA,1,NA,NA,NA,NA,NA,NA,NA,NA,NA,NA,NA,NA,1,NA,NA,1,NA,NA,NA,0,NA,0,NA,0,0,0,0,NA,NA,NA,NA,NA,1,NA,NA,0,NA,NA,NA,NA,NA,0,0,1,NA,NA,0,1,NA,NA,NA,NA,1,NA,1,0,NA,NA,NA,NA,NA,0,1,0,1,NA,0,NA,NA,NA,NA,1,NA,0,NA,NA,0,NA,NA,NA,NA,NA,NA,NA,0,1,NA,0,1,NA,NA,NA,1,0,NA,NA,1,1,1,NA,NA,0,1,NA,1,NA,NA,NA,NA,NA,NA,NA,0,NA,NA,1,NA,NA,NA,NA,0,NA,1,1,NA,NA,NA,NA,NA,NA,NA,0,NA,0,1,NA,NA,0,NA,NA,NA,NA,NA,NA,NA,NA,NA,NA,NA,NA,NA,NA,NA,NA,NA,0,NA,0,NA,NA,NA,NA,0,NA,NA,NA,NA,NA,NA,NA,NA,1,NA,NA,NA,NA,NA,NA,NA,NA,NA,NA,NA,NA,NA,NA,0,NA,NA,NA,NA,NA,NA,NA,NA,1,NA,NA,NA,0,NA,NA,NA,NA,NA,NA,NA,0,NA,NA,NA,NA,NA,NA,NA,NA,NA,NA,NA,NA,NA,NA,NA,NA,NA,NA,NA,0,NA,NA,NA,NA,NA,NA,NA,NA,NA,NA,1,NA,NA,NA,NA,0,NA,NA,NA,NA,NA,NA,0,NA,NA,NA,NA,NA,NA,NA,NA,NA,NA,NA,NA,NA,NA,NA,NA,NA,NA,NA,NA,NA,NA,0,NA,NA,NA,NA,NA,NA,NA,NA,NA,NA,NA,NA,NA,1,NA,NA,NA,0,NA,NA,NA,NA,NA,NA,0,NA,NA,NA,1,NA,NA,1,NA,NA,NA,NA,NA,NA,NA,NA,NA,NA,NA,1,NA,NA,0,0,NA,NA,NA,NA,NA,NA,NA,NA,NA,NA,NA,NA,NA,NA,NA,NA,NA,NA,NA,NA,NA,NA,NA,1,NA,NA,NA,0,NA,1,NA,NA,NA,NA,NA,NA,NA,0,NA,NA,NA,NA,NA,NA,NA,NA,NA,NA,NA,NA,NA,NA,NA,NA,NA,NA,NA,NA,0,NA,NA,NA,0,1,NA,NA,NA,NA,NA,NA,NA,1,NA,1,NA,1,NA,NA,NA,NA,NA,NA,NA,1,NA,NA,NA,NA,NA,NA,NA,NA,NA,NA,NA,1,1,NA,NA,NA,NA,NA,NA,NA,NA,NA,NA,0,NA,NA,0,NA,NA,0,NA,NA,NA,NA,1,NA,NA,NA,NA,NA,NA,NA,NA,NA,NA,NA,1,NA,NA,NA,0,NA,NA,NA,NA,NA,NA,NA,NA,NA,NA,NA,NA,NA,NA,NA,NA,NA,NA,NA,NA,NA,NA,NA),

# number unbanded adults found each breeding season from 2007/08 to 2016/17

# the “NA” corresponds to the initial release in March 2007

n.unb=c(NA,0,0,0,0,1,4,7,10,7,8)

)

**Appendix 4.** Means and credible limits (CL) for parameters in the full model and reduced models of fecundity and survival of North Island robins at Tawharanui Regional Park. These estimates are based on all 9 years of data with uninformative priors.

| Parameter ^a^ | Full Model | | | Reduced Model ^b^ | | |
| --- | --- | --- | --- | --- | --- | --- |
|  | mean | 2.5% CL | 97.5% CL | mean | 2.5% CL | 97.5% CL |
| a.f | 1.52 | 1.24 | 1.78 | 1.52 | 1.30 | 1.72 |
| b.d.f | -0.48 | -1.04 | 0.09 | -0.48 | -0.86 | -0.08 |
| sd.fem.f | 0.21 | 0.09 | 0.33 | 0.21 | 0.06 | 0.34 |
| sd.yr.fem. | 0.10 | 0.00 | 0.30 |  |  |  |
| a.phi | -1.05 | -1.98 | -0.01 | -1.44 | -1.95 | -0.93 |
| b.age.phi | 2.12 | 0.09 | 4.63 | 2.70 | 2.16 | 3.26 |
| b.bfl | 2.65 | 0.53 | 5.22 | 1.82 | 0.37 | 2.87 |
| b.d.juvphi | -0.83 | -2.62 | 0.95 |  |  |  |
| b.sex.adphi | -0.29 | -5.59 | 2.98 |  |  |  |
| b.trans | 2.70 | -1.01 | 14.11 |  |  |  |
| sd.yr.adphi | 0.25 | 0.01 | 0.84 |  |  |  |
| sd.yr.juvphi | 0.58 | 0.17 | 1.23 | 0.66 | 0.30 | 1.30 |
| a.p | 2.29 | 1.73 | 3.04 | 2.22 | 1.86 | 2.64 |
| sd.yr.p | 0.49 | 0.03 | 1.69 |  |  |  |

^a^ a.f, intercept term for log of mean fecundity (number of fledglings per female); b.d.f, effect of density (birds/ha) on log fecundity; sd.fem.f, standard deviation for random effect of individual female on log fecundity; sd.yr.fem, standard deviation for the random effect of year on log fecundity; a.phi, intercept term for logit of probability of a juvenile surviving from fledgling to adulthood and staying at Tawharanui; b.age.phi, difference between logit annual adult female survival and logit juvenile survival; b.bfl, effect of being banded as a fledging on logit juvenile survival; b.d.juvphi, effect of density on logit juvenile survival; b.sex.phi, difference between males and females in logit adult survival; b.trans; difference between logit survival probability in the first 6 months after translocation versus later; sd.yr.adphi, standard deviation for random annual variation in logit adult survival; sd.yr.juvphi, standard deviation for random annual variation in logit juvenile survival; a.p, intercept for logit of re-sighting probability; sd.t.p standard deviation for random annual variation on logit re-sighting probability.

^b^ Fixed effects removed if 95% credible intervals included zero, random effects removed if 95% credible intervals centred near 0.
